# Undergraduate palliative care education in the United Arab Emirates: a nationwide assessment of medical school deans

**DOI:** 10.1186/s12909-021-02966-4

**Published:** 2021-10-09

**Authors:** Thana Harhara, Halah Ibrahim

**Affiliations:** Department of Medicine, Sheikh Khalifa Medical City, PO Box 51900, Abu Dhabi, United Arab Emirates

**Keywords:** Palliative care, End-of-life care, Undergraduate medical education, Middle East, United Arab Emirates

## Abstract

**Background:**

The provision of comprehensive, high quality palliative care (PC) is a global public health concern. In the United Arab Emirates (UAE), palliative medicine services are limited, and most patients in need of PC are treated in the acute hospital setting, where health professionals of all specialties provide treatment. Improving end-of-life care requires teaching medical students, residents, and other healthcare professionals about PC. The purpose of this study was to assess the current status of PC education in medical schools in the UAE, and to identify barriers to successful implementation of a PC and end-of-life curriculum.

**Methods:**

The authors conducted semi-structured interviews with deans from all medical schools in the UAE. Data were analyzed using qualitative content analysis.

**Results:**

All medical school deans in our study recognized the importance of inculcating palliative and end-of-life care into the undergraduate curriculum, but there was substantial variability in implementation, with opportunities for improvement. Barriers to the successful implementation of an undergraduate PC curriculum include (1) lack of student awareness and interest in PC, (2) inconsistent clinical exposure to PC, (3) lack of specialized PC faculty, (4) limited clinical facilities for PC training, (5) lack of a multidisciplinary approach to PC education, and (6) cultural barriers to PC education.

**Conclusions:**

Understanding challenges to teaching PC in the undergraduate medical curriculum can help inform educational interventions to improve PC knowledge and skills for UAE medical students. Curricular and policy reform are necessary to educate a future generation of health professionals, who can provide high quality palliative care services to UAE patients and their families.

**Supplementary Information:**

The online version contains supplementary material available at 10.1186/s12909-021-02966-4.

## Background

The provision of comprehensive, high quality palliative care (PC) is a global public health concern. Over the next several decades, the world’s older population is expected to increase exponentially, with a corresponding surge in the incidence of cancer. In many Western countries, structured PC services have developed in parallel with the aging population. PC improves symptom control, helps to alleviate pain and suffering, improves quality of life, and increases patient and caregiver satisfaction [1]. Palliative medicine, as a discipline, has also broadened from its initial focus on terminal cancer care to include a wide spectrum of life-threatening illnesses. However, palliative medicine remains underdeveloped or non-existent in many parts of the world [1]. PC services in the Middle East are currently limited, with none of the countries approaching integration into mainstream health service provision [2]. This is especially concerning as a recent World Health Organization (WHO) mortality projection predicts an increase in the burden of serious health-related suffering through 2060 in all WHO regions, with the highest proportional increases (170%) expected in the eastern Mediterranean region [3]. As such, the number of individuals in the Middle East in need of palliative medicine will rise dramatically over the next forty years [3].

The United Arab Emirates (UAE) is a small, high-income nation in the Middle East. Over the past several decades, the country has experienced an aging population and increased prevalence of non-communicable and chronic diseases [4]. In addition, the prevalence of cancer in the UAE is high and rising [5]. In response, there has been significant national investment in healthcare resources over the past twenty years. However, PC services have lagged behind considerably. The first comprehensive government funded PC program in the UAE was established in Tawam Hospital in Al Ain in 2007 [6]. Within the private healthcare sector, dedicated PC services are offered at the American Hospital in Dubai, Mediclinic in Dubai and Burjeel Medical City in Abu Dhabi [6]. As a consequence of the limited PC services, most patients in need of palliative medicine are treated in the acute hospital setting, where health professionals of all specialties and training levels provide care. Therefore, improving end-of-life (EOL) care requires not only providing PC services to patients and families, but also on teaching medical students, residents, and other healthcare professionals about palliative medicine. Medical schools should be aware that their newly graduated doctors will care for patients with terminal illnesses. It is estimated that in the first year of practice, a doctor in the United Kingdom may treat approximately 160 dying patients [7]. Although there is limited published data for the UAE, cancer is the third leading cause of death in the country, and as most deaths occur in the hospital, it is likely that UAE physicians care for a large number of terminal patients [8]. Despite its importance for future physicians, PC concepts are largely omitted from university curricula worldwide [9, 10]. Even in countries in which PC is mandated as part of the health education curriculum, multinational studies report inadequate education and training in EOL care, leaving a majority of medical graduates feeling poorly prepared for these tasks [9, 10].

Studies in the Middle East have also documented deficiencies in PC training. For example, in a cross-country survey of 700 healthcare professionals in the Middle East, 86% desired additional knowledge of PC [11]. A survey of medical residents in Saudi Arabia found that over 90% denied receiving any training in PC, and half expressed negative views toward PC [12]. Currently, regulatory boards in the UAE do not explicitly mandate training in EOL care. To our knowledge, how UAE medical schools incorporate PC teaching into their curricula has not been looked at systematically on a national level. The purpose of this study was to assess the current status of PC education in medical schools in the UAE, and to identify barriers to successful implementation of a PC and EOL curriculum. Understanding challenges to teaching PC in the undergraduate medical curriculum can help inform educational interventions to improve PC knowledge and skills for UAE medical students.

## Methods

### Interview guide

The theoretical underpinnings for this study were based upon EOL care domains identified by Schaefer and colleagues, developed from a national survey in the United States to define essential PC competencies for medical students and residents [13]. They include (1) palliative care principles and practice, (2) fundamentals of pain and symptom management, (3) psychosocial, spiritual and cultural needs, (4) communication, and (5) terminal care and bereavement [13]. The initial semi-structured interview guide (attached) was developed based on these domains by one of the authors (TH), who has expertise in medical education and palliative care. The instrument was piloted for process, clarity and comprehension on three clinician-educators, who were medical school faculty. These individuals were knowledgeable about the medical school curriculum, the clinical learning environment, and medical student patient care activities in the teaching hospitals. Semi-structured questions sought discrete information (courses taught, number of hours), but enabled the respondents to offer additional details. We aimed to identify any EOL care domains, as described by Schaefer and colleagues, that were included in the curriculum [13]. Other questions were open-ended and designed to promote reflective analysis of the curriculum’s strengths and weaknesses. Finally, we elicited advice for curricular reform and policy change in PC education. Data collection and analysis occurred concurrently, and led to iterative adjustments of the interview guide.

### Setting and Participants

There are currently eight accredited medical schools in the UAE (Table 1). Khalifa University is the only school in the country that follows a postgraduate medical curriculum, whereby the students have at minimum obtained a Bachelor’s degree prior to medical school matriculation. The remaining schools offer foundational pre-medical courses in the first 1 to 2 years. Although duration of education varies, all of the schools must meet similar accreditation standards, with uniform admission and graduation requirements, and have similar curricula, with education in the basic sciences, followed by clinical rotations in the teaching hospitals. Study participants included a purposive sample of deans from all eight of the medical schools in the UAE. We wanted to ensure representation of individuals who had extensive knowledge of the medical school curriculum. These individuals are involved in curriculum design and implementation, and can advocate for the development and inclusion of a PC curriculum. Participants were identified through an Internet-based search and/or personal knowledge, and were invited via an e-mail that stipulated our intentions to interview them about the school’s PC curriculum. All respondents provided informed consent. Participation was voluntary, and we did not offer any incentives. The study was approved by the Sheikh Khalifa Medical City institutional review board in Abu Dhabi, UAE (RS-564).

**Table 1:** Characteristics of Medical Schools in the United Arab Emirates

University	Emirate	Type	Year of Establishment	Duration of Education (years)
Ajman University College of Medicine	Ajman	P	2018	6
Dubai Medical College for Girls	Dubai	P	1986	5
Gulf Medical University	Ajman	P	1998	5
Khalifa University	Abu Dhabi	G	2018	4
Mohammed Bin Rashid University	Dubai	P	2016	6
Ras Al Khaimah Medical and Health Sciences University	Ras Al Khaimah	G	2006	5
United Arab Emirates University	Abu Dhabi	G	1986	6
University of Sharjah	Sharjah	P	2004	6

Abbreviations: G = Government; P = Private

### Data Collection

From June 2020 through February 2021, both authors conducted eight semi-structured telephone interviews with deans from all of the medical schools in the UAE. The interviews were conducted in English, and lasted approximately 30 to 40 min each. We accomplished member checking in real time, with the interviewer frequently restating participants’ responses to ensure intended meaning. The interviews were audio-recorded with the participants’ permission, and transcribed verbatim by professional transcriptionists. The transcripts were checked against the recordings for accuracy, and anonymized prior to data analysis.

### Data analysis

We performed all data management, coding and analysis manually. Through an initial process of familiarization, we read through all of the de-identified transcripts to gain an overall view of the responses. To identify barriers to PC curricular development, we performed a qualitative analysis using a conventional content analysis approach to identify recurring concepts related to implementing a PC curriculum [14]. Each author independently performed a line-by-line open coding on the transcripts and initial codes were recorded. Themes were categorized through constant comparison in the process of refining the categories. Discrepancies were resolved through in-depth conversations. Through consensus, the coding scheme was established and then applied to all transcripts. We reached theoretical saturation for thematic content after reviewing five transcripts, but we analyzed and reported the data from all interview transcripts. Trustworthiness of results was reached by having two of the authors (HI, TH) participate in the iterative data analysis and by conducting member checking. To enhance credibility, typed transcripts were sent to four of the participants (50%) for confirmation and further input. In addition, upon completion of data analysis, we sent an email to all of the deans describing the findings of the study, to allow the participants to comment on the accuracy of the results of the qualitative analysis. We used the Standards for Reporting Qualitative Research (SRQR) checklist when writing our report [15].

## Results

All university deans agreed that competence in PC was an essential component in undergraduate education. One dean explained:

“The population is aging and they [medical students] will be encountering more and more people with these issues, and the technology is advancing, and life expectancy is increasing. They will be encountering people at the end-of-life, who will require complex care. It is a competency which every medical student must know before they graduate. Period.”

Despite its importance, only one of the private medical schools offered a dedicated palliative care course within the curriculum, and none offered a mandatory clinical rotation in PC. Instead, PC topics were mostly integrated into existing courses, rather than being taught as a separate module or course within the undergraduate curriculum. The number of hours allocated to PC content differed widely; particularly since PC content embedded in other courses could not always be clearly identified and quantified. One dean described:

“We review pain management in the Neuroscience module. There are components on communication in difficult situations in Introduction to the Practice of Medicine course, and I think some on the Foundation of Clinical Medicine course. And there are components on end-of-life ethical dilemmas in the Bioethics course. We don’t have a palliative care course, but it’s all embedded within these other modules.”

Another dean described his concerns with this approach:

“The disadvantage of this is that when you go back and ask the students have you done this [PC], they will tell you ‘no.’ So, what’s the point? This is my problem. It’s done in bits and pieces, and it gets lost in the whole scenario…You could see that on a piece of paper, it looks okay, but then from the student point of view, they may or may not give you a positive response.”

Pain management and EOL communication skills were the most frequently taught topics, followed by the ethics of terminal care. Content was most often delivered by lecture, followed by case-based learning, workshops and problem-based learning sessions. None of the universities have academic faculty positions for palliative medicine. Clinical and bedside teaching in PC is inconsistent and often led by faculty, who are not PC specialists. Schools do not routinely integrate other professions, including nurses, social workers and faith-based leaders, into the teaching teams.

The deans identified several barriers to PC education, described below and listed in Table 2.

**Table 2:** Barriers to Implementing a Palliative Care Curriculum in Undergraduate Medical Education^a^

Barriers	
1. Lack of medical student awareness and interest in PC 2. Inconsistent clinical exposure to PC 3. Lack of specialized PC faculty 4. Limited clinical facilities for PC training 5. Lack of a multidisciplinary approach to PC 6. Cultural barriers to PC education	

^a^as identified by UAE medical school deans

PC = palliative care

### Lack of student awareness and interest in PC

Students generally lacked awareness of PC as a discipline, and even when available, did not select PC electives. As one dean noted:

“That is because it’s a chicken and egg story. What is first? If they are exposed, then they may recognize that there is this area with great potential, and career counseling is important. We have to have palliative care experts coming in, giving talks to the students on a periodic basis. So, that’s how they’re going to develop interest.”

Another dean concurred:

“Elective options are available, but we haven’t had anyone actually asking for palliative care. It’s the lack of awareness. They do need to be more aware that the population is aging and palliative medicine is a specialty.”

### Inconsistent clinical exposure to PC

The participants recognized that clinical exposure in palliative medicine was limited and inconsistent. One dean admitted: “They do clerkships in hospitals. How much they learn about end-of-life care? I don’t think they learn a significant amount of that.” Another dean agreed: “Of course, they see patients, but based on opportunities, not planned…. Some of them see palliative patients; some of them don’t.”

### Lack of specialized PC faculty

Bedside teaching in end-of-life care was not offered on a regular basis, because universities struggled with recruiting faculty with specialized PC background. One dean explained:

“Definitely, I think PC is very important to include in our curriculum, but how do we do that with the current resources we have and the challenges of clinical exposure? Lack of expertise as well. We need people who are experts in communication- end-of-life communication. We need communication integrated with the care, and that’s what we’re lacking, along with pain management experts. We would love to have this. This really needs a proper plan.”

### Limited clinical facilities for PC training

An additional major curricular challenge was finding a sufficient number of specialized institutions for clinical rotations. Most of the palliative care centers in the UAE are located in private healthcare facilities, and are not often involved in medical training. This is a notable loss of expertise; university officials are working to establish alliances and institutional partnerships. One dean described:

“I think this is a major hurdle actually that should be taken up by the Ministry [of Health]. We need the healthcare facilities, which are equipped and have enough resources and expertise to teach palliative care in the undergraduate and postgraduate curriculum…. So, if you are now advocating the incorporation of a course in palliative care in my curriculum, I will see all the problems with this. How will I implement? Who will implement? Where will we implement? So, everywhere, there will be blank spaces, no expertise, no healthcare facility and what will I do?”

### Lack of a multidisciplinary approach to PC education

Several deans cited the inability to offer a “holistic” or multidisciplinary approach to PC as a challenge. None of the medical schools offered sessions related to EOL care by nurses, allied health professionals or faith-based providers. One dean commented:

“I am aware of only one palliative care physician who works for [the hospital] at the moment…. But we cannot link it to the MDT [multidisciplinary team] approach that we would have in terms of managing a patient towards the end of their life. These other services are not available for the students to observe.”

### Cultural barriers to PC education

Only one of the deans, from a private medical school, reported cultural barriers to clinical teaching in PC. These included patient and family resistance to student involvement in EOL care, and the lack of hospital policies regarding EOL care provision. He explained:

“But here [in the UAE], it’s absolutely different. The students are not, not even the residents, they are not called to communicate with patients about sensitive issues like breaking bad news, or that sort of thing…. I don’t think any curriculum in this region will allow medical students to talk about bereavement to the patients and their families. It’s such a sensitive issue that the senior physician should be having this conversation. I strongly support that we should modify our curriculum and policies and procedures to allow the students to have at least a role in such a conversation, in the presence of treating physicians.”

Many of the deans reported that they were actively working to develop alliances and university partnerships to improve clinical exposure in PC. One described:

“We have a relationship with our clinical affiliate, so that we could have joint appointments. We also have adjunct faculty appointments. So, as part of the PC curriculum, it’s a given that we would be working very closely with the PC specialist in the hospital. But, if we feel that we need more of their time, then we have models for joint appointments.”

## Discussion

In this study, we interviewed deans of all UAE medical schools to understand how palliative care was integrated into their curriculum and potential barriers to effective PC and EOL education. Although all respondents felt that PC exposure and training was an essential component of medical training, there was substantial variability in the content, mode of delivery and time allocated to palliative medicine topics, with inconsistent and fragmented exposure to PC patients in the clinical years.

The lack of a dedicated palliative medicine course within the undergraduate curriculum is a concern. Most of the topics are embedded within other courses, and primarily covered under the umbrella of communication skills, ethics or professionalism. Further, several universities present these topics through workshops and didactics entitled “end-of-life care” and “death and dying.” Studies have shown that medical trainees do not understand the difference between PC and providing hospice or EOL care to terminal patients [16]. PC can be provided in conjunction with life-prolonging or curative modalities, and has been shown to improve the quality of life of patients with serious illness, regardless of whether or not that illness is terminal. As such, this confusion can become an impediment to the provision of PC services to non-terminal patients [16]. This also likely contributes to limited student awareness of PC as a distinct medical specialty. The lack of formal PC training in the undergraduate medical curriculum has been cited as a barrier to the development of the discipline [17]. Therefore, formally incorporating a dedicated PC course into medical school education may also serve to advance the status of PC development in the UAE. We are encouraged that some universities are in the process of developing integrated and longitudinal PC curricula. This is supported by multiple studies that have shown that longitudinal teaching, throughout the continuum of medical education, is the most effective method of improving medical students’ confidence and skills in EOL care [18].

Both didactic and clinical exposures to palliative medicine are important components in medical training. Lecture-based teaching alone, which was reported to be the primary mode of PC teaching by our respondents, may not be as effective as direct experience in changing a student’s attitude toward EOL care. Studies have shown that clinical exposure during clerkship is the most critical factor positively impacting perceptions of EOL treatment [9, 10]. However, deans in our study described significant gaps in clinical exposure to PC. This is consistent with global studies, in which medical students frequently reported being excluded from meeting terminal patients, or actively avoided dealing with palliative patients [19]. In the United States, medical residents appreciated the importance of observing a faculty member discuss EOL care with patients, but most admitted that they were never observed or received feedback on their own EOL communication skills [20]. In a study of 126 medical students in Lebanon, only 14% had the opportunity to observe a senior physician disclose bad news to patients [21]. This limited exposure and lack of feedback can lead to deficits in confidence, empathy and communication skills. A systematic review of PC education in United States medical schools noted improvement in medical student competency in EOL care, regardless of the teaching method [22]. Knowledge and skills were improved through either clinical rotations or multi-faceted interventions [22]. As such, we are encouraged by the commitment of university leadership to promote PC training, and confident that increased awareness and exposure will help educate a cadre of physicians with the knowledge, skills and confidence to better care for the country’s aging population and their anticipated PC needs.

It is notable that cultural issues were rarely identified as obstacles to PC provision or education by the university deans. There is a large body of literature describing cultural and religious objection to PC services in the UAE [2, 5]. The perceived lack of cultural barriers may be due to the limited student involvement in EOL care. It may also reflect the changing demographics of the multicultural UAE society. This is an important area for future study.

Our findings are consistent with reports from medical schools worldwide, which document deficits in PC and EOL education [9, 10]. The study adds to the literature by providing the perceptions and intentions of medical school deans, and identifying barriers they faced in providing PC education. We found a strong interest from UAE medical deans to integrate palliative medicine into the curriculum. Despite the intent, we believe that, ultimately, a national teaching framework for culturally competent and locally relevant PC should be adopted to standardize exposure and learning, and to improve the recognition of palliative medicine as an integral part of medical training. This is a long-term goal that will require the recruitment of PC specialists, who can develop and deliver the content, and role model the care. Studies have shown that EOL teaching by PC specialists improves student self-efficacy in PC [23]. Recruitment efforts must coincide with curricular and policy reform. Studies suggest that the following interventions are feasible and actionable, and can improve PC and EOL training for medical students in the UAE and the region (Table 3). First, formal palliative care/EOL curricula should be mandatory in undergraduate medical training, and should cover the core PC competencies for medical students [13]. To accomplish this, faculty development will be necessary. Professional development programs in pain management, EOL symptom management, and EOL communication skills should be available for all medical school faculty. Further, clinical experience in PC should be expanded, and become a mandatory component of training. Partnership with PC centers in the private sector will be necessary to provide students with exposure to PC and EOL care in dedicated inpatient units and outpatient PC clinics. Medical schools can also partner with professional societies. Organizations, such as the Middle East Cancer Consortium and the newly developing Emirates Palliative Care Society, can provide expertise and resources for the schools. Students can become involved in webinars and conferences; thereby, increasing awareness and building knowledge and confidence. Finally, students need training in personal reflection on their experiences with patient death, as well as structured debriefing that explores effective coping strategies. It is important to provide a safe environment for students to discuss the emotions inherent in death. Without appropriate support, the learning experience can be negative [24].

**Table 3:** Implications for Curricular and Policy Reform

Pertinent Findings from the Study	Implications for Curricular and Policy Reform
Most UAE medical schools do not offer dedicated PC courses within the curriculum.	Formal palliative care/EOL curricula should be mandatory in undergraduate medical training, and should cover the core PC competencies for medical students. Professional development programs in pain management, EOL symptom management, and EOL communication skills should be available for all medical school faculty.
There is inconsistent clinical exposure to PC patients.	Clinical experience in PC should be expanded, and become a mandatory component of training. Partnership with PC centers in the private sector can provide students with exposure to PC and EOL care in dedicated inpatient units and outpatient PC clinics outside of the acute care setting.
Medical students lack awareness and interest in PC.	Medical schools can partner with professional societies. Organizations, such as the Middle East Cancer Consortium and the newly developing Emirates Palliative Care Society, can provide expertise and resources for the schools. Students can become involved in webinars and conferences; thereby, increasing awareness and building knowledge and confidence.
UAE medical schools lack a multidisciplinary approach to PC and EOL care	Medical schools should routinely integrate other professions, including nurses, social workers and faith-based leaders, into the teaching teams.

EOL = end-of-life; PC = palliative care

Our study has several limitations. First, a small number of individuals was interviewed, though deans from all medical schools in the country were included. We believe our findings represent the current PC curriculum being taught to the country’s future physicians. Next, we could document the existence of PC content, but could not assess the quality of the clinical or educational service being provided. Finally, the medical student perspective is missing. Despite these limitations, the study offers important insights into identifying, and potentially overcoming, barriers to teaching PC as part of the undergraduate medical curriculum. Although the study was conducted in the UAE, we believe our findings can help inform curricular reform worldwide. Future studies are needed to explore the impact of PC curricula on UAE medical students, and on their care of palliative patients and their families.

## Conclusion

As UAE medical schools adapt to an aging population and increased need for PC services, increased PC training is paramount. All medical school deans in our study recognized the importance of inculcating EOL care into the undergraduate curriculum, but there was substantial variability in implementation, with opportunities for improvement. Curricular and policy reform are necessary to educate a future generation of health professionals who can provide quality, culturally sensitive PC services to UAE patients and their families.

## Supplementary Information

**Additional file 1.**

## Data Availability

Data can be provided by corresponding author upon reasonable request.

## Abbreviations

EOL: End-of-life
PC: Palliative care
UAE: United Arab Emirates
WHO: World Health Organization
