# Supplementary material for: Undergraduate palliative care education in the United Arab Emirates: a nationwide assessment of medical school deans
Source: BMC Med Educ. 2021 Oct 9;21:526. doi: 10.1186/s12909-021-02966-4 (PMC8502263; doi:10.1186/s12909-021-02966-4)
Supplement: Supplementary file 1 — Additional file 1. [file 12909_2021_2966_MOESM1_ESM.docx]

**
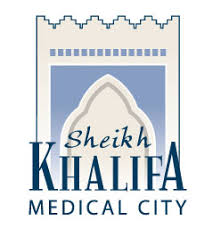
Palliative Care Curriculum in Undergraduate Medical Schools in the** **UAE**

**Interview Guide**

For the purpose of our conversation, **palliative care** is defined as an approach that improves the quality of life of patients and their families facing problems associated with life-threatening illness, through the prevention and relief of suffering (WHO).

**End of Life care** is defined as care for people in decline who are deemed to be terminal or dying in the foreseeable future (CIHI).

**Palliative Care Teaching and Resources**

1. Are your medical students given any formal courses or teaching on palliative care and end-of-life care?

Why/why not?

Who/what led to the introduction of the teaching?

1. Are your medical students given any courses that do not focus primarily on palliative care but contain components that address palliative /end-of-life care (example geriatrics, anesthesia, ethics, or communication skills)?

Can you give me examples?

1. Which teaching formats are used for palliative education for your medical students?

- *Formal Lectures*
- *Case-based learning*
- *Small-group discussions*
- *Large-group discussions*
- *Problem-based learning*
- *Computer-based learning*
- *Clerkships or clinical rotations*
- *Simulation or professional patients*
- *Other, please explain*
- *None*

1. How many hours in the curriculum are dedicated to palliative care/end of life care?
2. Which palliative medicine topics are covered?

- *Introduction to palliative care*
- *Pain management*
- *Non-pain symptom management*
- *End-of-life care*
- *Bereavement and psychosocial support*
- *Code status or advanced care planning*
- *Psychological support*
- *Spiritual support*
- *Ethical and legal issues*
- *Communication skills in end of life*
- *Teamwork and/or interprofessional care in end of life*
- *Medical professionalism and end of life care*

1. Are they covered explicitly as part of an End-of-Life (EOL) and palliative care curriculum?
2. Does your medical school have academic faculty positions for palliative care?

**Palliative Care Experience**

1. Do your medical students participate in a mandatory rotation in palliative care?

How long is the rotation?

1. Do your medical students participate in an elective rotation in palliative care?

How long is the elective rotation?

What percentage of students takes this elective rotation?

1. Are your students involved in taking care of terminally ill patients or patients at EOL?
2. How much exposure do you think your students have to such patients?
3. Is there a mechanism for your students to discuss/debrief their interactions with EOL patients?

Please describe. When? With whom?

**Evaluation**

1. Are your medical students evaluated on their knowledge in EOL/palliative care?
2. How are they evaluated?

**Learning Needs and Barriers**

1. How important is it to your institution that medical students learn to provide care for dying patients?
2. Would you be supportive of integrating end-of-life care into your curriculum?

Why?

How would you integrate it?

1. In your opinion, what are potential barriers to teaching end of life care in undergraduate medical education in the UAE?

- *Lack of faculty*
- *Lack of time*
- *Lack of curriculum*
- *Low priority*
- *Lack of clinical services, ex. Palliative care service*

**Thank you for your time. Do you have anything else you would like to share?**
